# Preliminary study investigating the role of estimated glomerular filtration rate, proteinuria and hypertension to inform on chronic kidney disease after acute kidney injury

**DOI:** 10.1111/jsap.70046

**Published:** 2025-12-29

**Authors:** L. P. Cole, L. Pelligand, R. Jepson, K. Humm

**Affiliations:** ^1^ Department of Clinical Science and Services Royal Veterinary College London UK

## Abstract

**Objectives:**

To assess chronic kidney disease in dogs after azotaemic acute kidney injury utilising serum creatinine, symmetric dimethylarginine and estimated glomerular filtration rate.

**Materials and Methods:**

Client‐owned dogs hospitalised for azotaemic acute kidney injury (T0) were evaluated at discharge (T1), 3 months (T2) and 12 (T3) months with serum creatinine and symmetric dimethylarginine measured. In non‐azotaemic dogs (serum creatinine <145 μmol/L) at T1 and T2, glomerular filtration rate was estimated by iohexol clearance. Acute kidney injury grade and chronic kidney disease stage were determined according to International Renal Interest Society guidelines. Non‐azotaemic dogs were considered to have kidney dysfunction if glomerular filtration rate was reduced ≥20% below the mean of the body weight category.

**Results:**

Fifteen dogs with azotaemic acute kidney injury were recruited. At T0 peak, acute kidney injury grade was III (*n* = 4), IV (*n* = 8) and V (*n* = 3). At T1, 10/15 dogs remained azotaemic. At T2, 3/15 dogs remained azotaemic; this persisted in 2/3 dogs at T3. One dog was euthanised prior to T3 due to progression of azotaemia (stage 4). Based on glomerular filtration rate assessment, 4/12 and 5/12 non‐azotaemic dogs had evidence of kidney dysfunction at T2 and T3, respectively. Ten out of 15 dogs were classified as International Renal Interest Society chronic kidney disease stage 1 and 4/15 dogs were stage 2 and 1/15 dog that did not survive to T3 was stage 4.

**Clinical Significance:**

Persistent azotaemia occurs infrequently in dogs surviving beyond 3 months after acute kidney injury. Estimated glomerular filtration rate may identify continued kidney dysfunction in non‐azotaemic dogs.

## INTRODUCTION

Acute kidney injury (AKI) is commonly encountered in dogs, with outcomes ranging from full renal recovery to fulminant kidney failure necessitating euthanasia or renal replacement therapy. A proportion of patients that survive the AKI episode are left with azotaemic chronic kidney disease (CKD) (Vaden et al., 1997). Using serum creatinine (SCr) measurement at varying time points, previous studies report an occurrence of azotaemic CKD post AKI of 50% to 61% in dogs and cats (Hetrick et al., 2022; Vaden et al., 1997; Worwag & Langston, 2008). Latest International Renal Interest Society (IRIS) guidelines indicate that all dogs that recover from an AKI should be considered to have CKD even when returning to a non‐azotaemic state due to increased risk for intrinsic kidney injury (Segev et al., 2024). In dogs with naturally occurring AKI where SCr was measured ≥3 months after an AKI episode, the majority were classified as IRIS CKD stage 1 and the rest as CKD stage 2, but normalisation of SCr was not associated with long‐term survival (Bar‐Nathan et al., 2022; Ioannou et al., 2024). None of these studies utilised standardised time points for creatinine measurement post AKI, nor was there assessment of factors that may be associated with increased risk of persistent azotaemic CKD.

Measurement of glomerular filtration rate (GFR) is the gold standard for assessment of kidney function and GFR estimation using serum iohexol clearance is clinically useful in diagnosing canine CKD before the onset of azotaemia (McKenna, Pelligand, Elliott, Walker, & Jepson, 2020). Furthermore, symmetric dimethylarginine (SDMA) may be a more sensitive marker than creatinine for detecting a ≥40% decrease in GFR (Hall et al., 2016; McKenna, Pelligand, Elliott, Cotter, & Jepson, 2020; Nabity et al., 2015). The modified IRIS guidelines (Cowgill, 2023) now include SDMA in their CKD staging system, and dogs with persistently elevated SDMA concentrations (>18 μg/dL) are classified as stage 1 CKD.

In people, it has been demonstrated that AKI recovery can take weeks to months (Kellum et al., 2012). The human consensus statement from the Acute Disease Quality Initiative has defined the diagnosis of CKD after AKI as the persistence of kidney disease for >90 days (Chawla et al., 2017; Kellum et al., 2012). Based on estimated GFR and SCr concentrations, CKD is common in people after even mild AKI (Horne et al., 2017). In people, significant persistent proteinuria has been identified as a risk factor for the progression of CKD (Hsu et al., 2020). The only veterinary study assessing GFR post AKI is a canine experimental model of AKI (Finco, 2004). In this study, all dogs had decreased GFR during the 16‐month follow‐up period and those dogs with systemic hypertension (SH) had significantly decreased GFR and elevated UPCR compared to normotensive dogs. SH has been previously reported to occur in 75% to 81% of dogs with community‐acquired AKI, although it has not been associated with survival to discharge and the effect on longer term outcomes is unknown (Cole et al., 2020; Geigy et al., 2011). Proteinuria has been reported in 78% of dogs with AKI (Cole et al., 2020) and has been shown to be associated with a worse short‐term outcome in dogs with AKI (Troìa et al., 2018) and worse long‐term outcomes in dogs with CKD (Jacob et al., 2005).

The primary aim of this study was to describe CKD post AKI using serum biomarkers (SCr and SDMA) and direct GFR estimation with iohexol clearance. The secondary aims were to assess the occurrence of persistent proteinuria and SH after AKI and to assess for any association between proteinuria, SH and CKD in a population of dogs presenting to a referral hospital with azotaemic AKI.

## MATERIALS AND METHODS

### Case selection

The study was approved by Royal Veterinary College Clinical Research and Ethical Review Board (URN 2020 1958‐3). Owner consent was obtained. Fifteen client‐owned dogs discharged from a university referral teaching hospital after diagnosis of azotaemic AKI were prospectively recruited between January 2021 and January 2022. Data were collected during hospitalisation (T0), at discharge (T1), 3 months (T2) and 12 months (T3) after discharge. Based on the institution treating approximately 100 dogs/year with community acquired AKI, an approximate in‐hospital mortality of 50%, an estimated 35% opt out and loss to follow‐up, we planned to recruit 15 dogs discharged after AKI.

AKI was diagnosed based on clinical signs and diagnostics consistent with AKI, that is, acute onset of azotaemia and graded based on the IRIS AKI grading guidelines (Cowgill, 2016; Segev et al., 2024). Dogs were classified as oligo/anuric based on urine output. Dogs with fluid responsive AKI (defined as SCr returning to reference interval within 48 hours of intravenous fluid therapy), clinical history and clinicopathological data and/or sonographic evidence consistent with CKD (e.g. small irregular kidneys, decreased cortico‐medullary distinction (Bragato et al., 2017)) or post‐renal azotaemia were excluded. Dogs that failed to survive 3 months after discharge were also excluded.

### AKI management

Treatment of dogs enrolled in the study including post‐discharge management was at the discretion of the attending clinicians. Signalment, body weight, cause of AKI (if known), systolic blood pressure (SBP) on admission and during hospitalisation, fundic examination results, urine output, presence of oligo/anuria, fluid overload and requirement for renal replacement therapy, SCr (on presentation, peak and on discharge), urine protein:creatinine ratio (UPCR), urine sediment examination and culture results were recorded. An active sediment was classified as presence of >10 red blood cells or >5 white blood cells per high power field (Meindl et al., 2019). The number of dogs with UPCR >0.5 and >2 was recorded.

Classification according to IRIS AKI grading was performed using the peak SCr concentration at T0 and SCr at T1 was used for IRIS AKI grade at discharge. Azotaemia was defined as a SCr >144.5 μmol/L (1.6 mg/dL), which represented the upper end of the laboratory reference interval (Royal Veterinary College Diagnostic Laboratory, London). SBP was measured with Doppler ultrasonic sphygmomanometry (VetBP Doppler, Burtons, Tonbridge, Kent). The number of dogs with a SBP ≥160 mmHg and ≥180 mmHg on admit and during hospitalisation was recorded (Acierno et al., 2018). SH was defined as dogs treated with anti‐hypertensive therapies and details of anti‐hypertensive medication administered were recorded. Leptospirosis was diagnosed based on blood or urine PCR results and/or microscopic agglutination titres results. A titre of a single Microscopic aagglutination test (MAT) ≥1:800 without recent (<12 months) history of vaccination, or when seroconversion in paired MAT titres (i.e. fourfold increase between the first and second samples) was used to diagnose leptospirosis (Sykes et al., 2011).

### Follow‐up

Dogs returned for repeat evaluation at 3 months (T2) and 12 months (T3) for staging and monitoring of CKD. SCr and SBP measurement and free catch urine analysis (urine specific gravity, UPCR and sediment examination) were performed at these time points. If dogs had an active sediment and clinical signs of a urinary tract infection, urine culture was performed and UPCR measurement was not performed until the resolution of clinical signs and an inactive sediment was obtained. Serum samples were submitted for SDMA quantification at a commercial laboratory (IDEXX laboratories, UK) and all other blood tests were submitted to the Royal Veterinary College Diagnostic Laboratory, London.

If the dog was non‐azotaemic (SCr ≤144.5 μmol/L (1.6 mg/dL)) at T2 and T3, GFR measurement was determined by intravenous iohexol clearance. After injection of 1 mL/kg iohexol a previously described protocol was followed by a single veterinarian for measurement of iohexol clearance (McKenna, Pelligand, Elliott, Walker, & Jepson, 2020). Serum iohexol concentration was measured using a validated high‐performance capillary electrophoresis method at a commercial laboratory, DeltaDot Ltd, using iopromide as an internal standard (Pelligand et al., 2015).

Absolute GFR was estimated from the serum clearance of iohexol by application of a compartmental model and canine‐specific correction formula, normalised to body weight in kilograms (Bexfield et al., 2008). Dogs were divided into four weight quartiles: Category 1: 1.8 to 12.4 kg, Category 2: 13.2 to 25.5 kg, Category 3: 25.7 to 31.6 kg and Category 4: 32.0 to 70.3 kg.

To determine relative GFR (% reduction in GFR), the estimated absolute GFR of each dog was compared with the expected mean GFR of their respective body weight categories: 2.89 mL/kg/min for Category 1, 2.4 mL/kg/min for Category 2, 2.16 mL/kg/min for Category 3 and 2.25 mL/kg/min for Category 4 (Bexfield et al., 2008). In the event that a dog’s body weight did not fall within the range of any of these categories, the dog was included in the category to which its body weight was closest. A GFR reduction of ≥20% below the mean of the body weight category, was used to classify dogs as having kidney dysfunction. Relative GFR was considered normal if it was above or within 20% below the mean of the body weight category (Finch et al., 2018; McKenna, Pelligand, Elliott, Walker, & Jepson, 2020).

IRIS guidelines were used for CKD staging of dogs; notably all dogs after AKI are classified as a minimum of CKD stage 1 with the specific stage determined by SCr and SDMA concentrations (Cowgill, 2023; Segev et al., 2024). CKD staging was based on SCr and SDMA concentrations at T2 and T3. Intraindividual differences in absolute GFR measurement (mL/kg/min), SCr and SDMA were determined based on T2 and T3 measurements (T2 GFR mL/kg/min – T3 GFR mL/kg/min).

### Statistical analysis

Descriptive statistics of the patient population and their clinicopathological data were performed. Frequencies are presented as fractions and percentages with corresponding 95% confidence intervals. Data were assessed for normality and reported as mean (standard deviation) and median (range). Statistical analysis was performed using commercial software (GraphPad Prism version 10.2.3 for Windows, GraphPad Software, Boston, Massachusetts USA). A mixed effect model with 2 fixed effects (time point and aetiology) and their interaction was used to detect any differences between measurement of SCr, SDMA and GFR (absolute and relative) comparing a diagnosis of leptospirosis and other AKI aetiology (binary predictor) at T2 and T3. A Bonferroni test was used for multiple comparisons. 95% confidence intervals and adjusted *P* values are reported with an alpha threshold of .05.

## RESULTS

### Case inclusion

**FIG 1:**
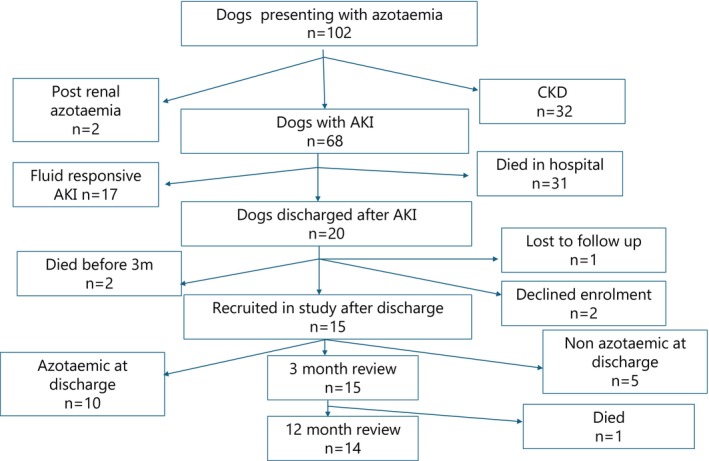
Flow chart of study participants from a population of azotaemic dogs presenting to a referral hospital.

One hundred and two dogs presented as emergencies with azotaemia during the study period (Fig 1). The pre‐determined sample size of 15 dogs was recruited after hospital discharge and all 15 dogs were evaluated at 3 months. One dog died between 3 and 12 months and therefore only 14 dogs were available for evaluation at 12 months.

The median age and weight of the dogs were 26 months (5 to 117) and 19.9 kg (5.1 to 76.3), respectively. There were 7/15 (47%; 95% CI 21% to 73%) female neutered, 5/15 (33%; 95% CI 11% to 61%) male neutered and 3/15 (20%; 95% CI 4% to 48%) male entire dogs. Thirteen of the dogs were pure breeds, of which 4/13 were Labrador retrievers. Nine dogs had a recent history of NSAID exposure, of which 8/9 (89%; 95% CI 51% to 99%) had been prescribed NSAIDs for therapeutic purposes. Of these, 6/8 (75%; 95% CI 35% to 97%) dogs had general anaesthesia (GA) and abdominal surgery, and the other two dogs had concurrent sedation. Four of the 15 (27%; 95% CI 7.8% to 55%) dogs were diagnosed with leptospirosis and in 2/15 (13%; 95% CI 1.7% to 40%) dogs, an aetiology could not be identified.

### AKI management

Serum creatinine concentrations and AKI grade at T0 and T1 are presented in Table 1. Four of the 15 (27%; 95% CI 7.8% to 55%) dogs were oligo/anuric of which three of the four underwent renal replacement therapy. SCr >144.5 μmol/L (>1.6 mg/dL) was present in 10/15 (67%; 95% CI 38% to 88%) dogs at T1.

**Table 1:** Median (range) serum creatinine, SDMA concentration, absolute GFR measurement in 15 dogs discharged after AKI and total number of dogs in each AKI grade during hospitalisation, categorised according to urinary output and CKD stage 3 to 12 months after discharge

Parameters	*n*	T0		*n*	T1	*n*	T2	*n*	T3
		Admit	Peak						
SCr	15			15		15		14	
μmol/L		574 (222 to 1244)	622 (222 to 1244)		189 (80 to 520)		111 (77 to 565)		99 (76 to 195)
mg/dL		6.49 (2.5 to 14.1)	7.03 (2.5 to 14.1)		2.14 (0.9 to 5.8)		1.26 (0.87 to 6.39)		1.12 (0.86 to 2.20)
SDMA		‐	‐	15	‐	15		14	
μg/dL							17 (8 to 43)		14 (12 to 30)
Absolute GFR		‐	‐	12	‐	12		12	
mL/kg/min							1.95 (1.15 to 2.96)		2.09 (1.19 to 2.93)
AKI grade	15	‐					‐	‐	‐
I			0		5				
II			0		5				
III			4 (O/A *n* = 0)		3				
IV			8 (O/A *n* = 2)		2				
V			3 (O/A *n* = 2)		0				
CKD stage		‐			‐		‐	14	
1									10
2									4
3									0
4									0

T0 During hospitalisation, T1 At discharge, T2 At 3 months, T3 At 12 months, SCr Serum creatinine, SDMA Symmetric dimethylarginine, GFR Glomerular filtration rate, AKI Acute kidney injury, O/A Oligo/anuric, CKD Chronic kidney disease, *n* Number of dogs

Admission and in‐hospital SBP are presented in Table 2. Four dogs had a recorded fundic exam and none had evidence of hypertensive ocular pathology. SH was diagnosed in 6/15 (40%; 95% CI 16% to 68%) dogs. Amlodipine was prescribed at a dose range of 0.1 to 0.2 mg/kg every 12 to 24 hours. Two of the six dogs (35%; 95% CI 4.3% to 78%) were discharged with anti‐hypertensive therapy. Ten of the 14 (71%; 95% CI 41% to 91%) dogs had UPCR >0.5 (Table 3). Two of the four (50%; 95% CI 6.8% to 93%) dogs with UPCR >2 had an active sediment. Initial urine culture was negative in all 15 dogs.

**Table 2:** Number of dogs in each blood pressure category at admission, during hospitalisation, and 3 and 12 months after discharge, kidney dysfunction defined as a serum creatinine of ≥144.5 μmol/L (1.6 mg/dL) or dogs with a GFR reduction of ≥20% below the mean of the body weight category

Blood pressure subcategory	T0 (*n* = 15)		T1	T2 (*n* = 15)		T3 (*n* = 13)	
	Admit	Peak					
				Kidney dysfunction (*n* = 7)	No kidney dysfunction (*n* = 8)	Kidney dysfunction (*n* = 6)	No kidney dysfunction (*n* = 7)
Normotensive (SBP <150 mmHg)	7	1	‐	4	4	4	4
Pre‐hypertensive (SBP 150 to 159 mmHg)	1	3	‐	2	3	1	3
Hypertensive (SBP 160 to 179 mmHg)	4	5	‐	0	1	1	0
Severely hypertensive (SBP ≥180 mmHg)	3	6	‐	1	0	0	0

T0 Hospitalisation, T1 Discharge, T2 3 months after discharge, T3 12 months after discharge, *n* Number of dogs, SBP Systolic blood pressure

**Table 3:** Urine protein: creatinine in hospital and 3 and 12 months after discharge

UPCR	T0 (*n* = 14)	T1	T2 (*n* = 15)		T3 (*n* = 14)	
			Kidney dysfunction (*n* = 7)	No kidney dysfunction (*n* = 8)	Kidney dysfunction (*n* = 7)	No kidney dysfunction (*n* = 7)
≤0.5	4	‐	7	8	7	6
>0.5 ≤ 2	6	‐	0	0	0	1
>2	4	‐	0	0	0	0

UCPR Urine protein:creatinine ratio, T0 Hospitalisation, T1 At discharge, T2 3 months after discharge, T3 12 months after discharge, *n*, Number of dogs

Kidney dysfunction is defined as a serum creatinine of ≤144.5 μmol/L (1.6 mg/dL) or dogs with a GFR reduction of ≥20% below the mean of body weight category

### Follow‐up

#### 3 months (T2)

The median SCr and SDMA concentration of all dogs at T2 are presented in Table 1. Three of the 15 (20%; 95% CI 4.3% to 48%) dogs had SCr >144.5 μmol/L (1.6 mg/dL) and two of the three (67%; 95% CI 9.4% to 99%) dogs had SDMA >18 μg/dL. The 12 dogs that had SCr ≤144.5 μmol/L (1.6 mg/dL) underwent GFR measurement using iohexol clearance. Median (range) absolute GFR measurement for all dogs at each time point is presented in Table 1. Four of the 12 (33%; 95% CI 10% to 65%) dogs had a decreased relative GFR (Table 4). SDMA was ≤18 μg/dL in all dogs that had GFR measurement. The median SDMA for dogs with normal relative GFR (*n* = 8) was 15 (8 to 17) μg/dL and the median SDMA for dogs with decreased relative GFR (*n* = 4) was 17 (12 to 17) μg/dL.

**Table 4:** Serial measurement of GFR, SCr and SDMA concentrations in 15 dogs discharged after acute kidney injury between 3 and 12 months based on aetiology

Dog	Suspected aetiology of AKI	SCr mg/dL (μmol/L)		SDMA μg/dL		Absolute GFR measurement (mL/kg/min)		Relative GFR (% reduction from mean of body weight category)	
		T2	T3	T2	T3	T2	T3	T2	T3
1	GA NSAIDS	111 (1.26)	93 (1.05)	17	13	2.03	2.4	−6.0%	0.0%^+^
2	Sedation/NSAIDS	93 (1.05)	84 (0.95)	16	12	1.98	2.25	−17.6%	−6.2%
3	GA/NSAIDS	143 (1.62)	138 (1.56)	17	20	1.32	1.44	−54.0%^*^	−50.2%^*^
4	Leptospirosis	85 (0.96)	83 (0.94)	15	15	1.92	2.64	−11.1%	+17.3%^+^
5	NSAIDS	88 (1.00)	88 (1.00)	8	15	2.96	2.56	+2.5%	−11.4%
6	Sedation/NSAIDS	132 (1.49)	87 (0.98)	17	12	1.35	1.36	−37.0%^*^	−43.6%^*^
7	Leptospirosis	69 (0.78)	76 (0.86)	13	14	2.61	2.93	+8.9%	+22.1%
8	GA NSAIDS	128 (1.45)	128 (1.45)	12	14	1.15	1.19	−48.7%^*^	−47%^*^
9	Leptospirosis	77 (0.87)	102 (1.15)	13	14	2.21	1.62	+2.4%	−24.8%^*^
10	Leptospirosis	81 (0.92)	96 (1.09)	11	14	2.27	2.66	−5.2%	+11.0%^+^
11	Unknown	106 (1.20)	117 (1.32)	17	15	1.93	1.93	−19.6%	−10.6%^+^
12	GA NSAIDS	118 1.33	111 (1.26)	17	13	1.44	1.25	−40.0%^*^	−42.0%^*^
13	GA NSAIDS	565 (6.39)	‐	43	‐			‐	‐
14	GA NSAIDS	145 (1.64)	150 (1.7)	18	13			‐	‐
15	Unknown	193 (2.18)	195 (2.21)	37	30			‐	‐

T2 3 months after discharge, T3 12 months after discharge, SCr Serum creatinine, GFR Glomerular filtration rate, NSAID Non‐steroidal anti‐inflammatory, GA General anaesthesia, SDMA Symmetric dimethylarginine

* A GFR reduction of ≥20% below the mean of the body weight category was used to classify dogs as having kidney dysfunction (McKenna, Pelligand, Elliott, Walker, & Jepson, 2020)

^+^ Denotes a dog that changed weight category for GFR measurement between 3 and 12 months

SBP categories at T2 are presented in Table 2. No dogs were receiving anti‐hypertensive treatment at T2. No dogs with SBP ≥160 mmHg had evidence of hypertensive retinopathy on fundic examination and the SBP normalised on subsequent follow‐up.

The number of dogs with proteinuria at T2 is presented in Table 3. The median USG for dogs with normal GFR (*n* = 7) was 1.035 (1.014 to 1.047) and for dogs with decreased relative GFR (*n* = 4) was 1.016 (1.014 to 1.022). Two dogs had an active sediment of which one had a positive urine culture that was treated and UPCR was re‐measured after completion of treatment, and this was the value included in the study analysis.

#### 12 months (T3)

One dog (dog 13 – Table 4) was euthanised between T2 and T3 due to progression of kidney disease and therefore 14 dogs were available for re‐evaluation at T3 (Fig 1).

The median SCr and SDMA, concentrations are presented in Table 1. Two dogs had SCr >144.5 μmol/L (1.6 mg/dL) of which one had SDMA >18 μg/dL. Both of these dogs had been azotaemic at 3 months (Table 4: cases 14 and 15). The median absolute GFR measurement for the 12 remaining dogs is presented in Table 1; 5/12 (52%; 95% CI 15% to 72%) had decreased relative GFR (Table 4), of which one had SDMA >18 μg/dL (Table 4: case 3). The median SDMA for dogs with normal relative GFR was 14 μg/dL (12 to 15) and the median SDMA for dogs with decreased relative GFR was 14 μg/dL (12 to 20).

SBP categories at the 12‐month re‐examination are presented in Table 2. No dogs were receiving anti‐hypertensive medication at T3. No dogs with SBP ≥160 mmHg had evidence of hypertensive retinopathy on fundic examination and the SBP normalised on subsequent follow‐up.

The number of dogs with proteinuria at T3 is presented in Table 3. The median USG for dogs with normal relative GFR (*n* = 7) was 1.038 (1.027 to 1.050) and for dogs with decreased relative GFR (*n* = 3) was 1.026 (1.017 to 1.029). Two dogs had an active sediment, one dog with UPCR >0.5.

### CKD staging

CKD staging is presented in Table 1; of the 10 dogs classified as IRIS CKD stage 1 based on history of AKI, three dogs had a reduction in relative GFR at 3 and/or 12 months. Two of the four (50%; 95% CI 15% to 85%) dogs with stage 2 CKD were non‐azotaemic and both dogs had decreased relative GFR (Table 4: cases 3 and 8).

### Serial SCr, SDMA and GFR measurement

**FIG 2:**
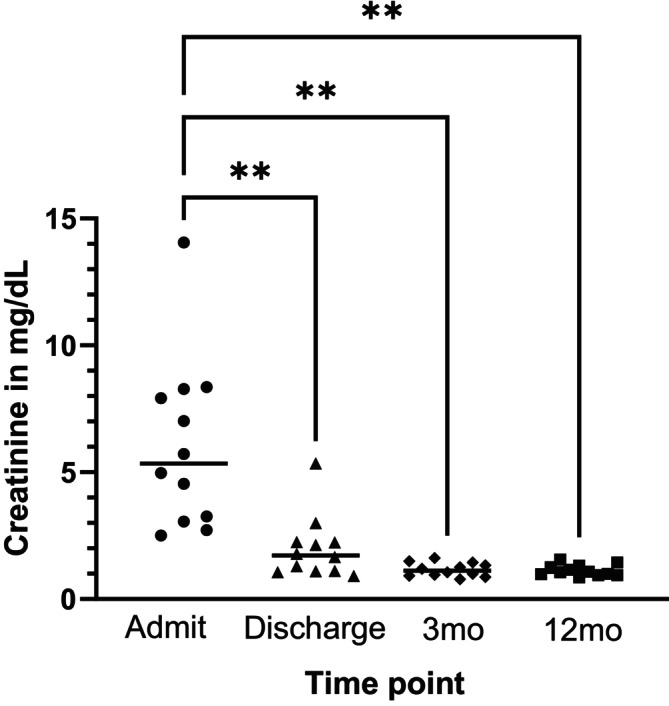
Change in serum creatinine (mg/dL) over time in 12 dogs after AKI where glomerular filtration rate measurement was performed. Data are presented as individual data points and median (horizontal line); ** indicates a significant difference between each time point with *P* values ranging between .002 and .005. Mo Months.

**FIG 3:**
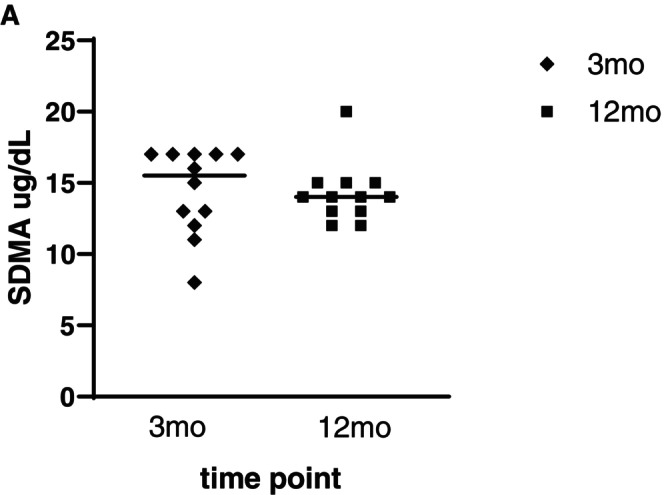
Change in SDMA (μg/dL) concentration over time in 12 dogs after AKI where glomerular filtration rate measurement was performed. Data are presented as individual data points and median (horizontal line). SDMA Symmetric dimethylarginine, Mo Months.

The median intraindividual difference in SCr and SDMA between T2 and T3 was 0.88 μmol/L, −44 to +25 (−0.01 mg/dL, −0.5 to +0.28) and + 0.5 μg/dL (−5 to +7), respectively. SCr and SDMA decreased in 6/14 (43%; 95% CI 21% to 68%) and 5/14 dogs (36%; 95% CI 16% to 61%), respectively, between T2 and T3 (Figs 2 and 3, Table 4).

**FIG 4:**
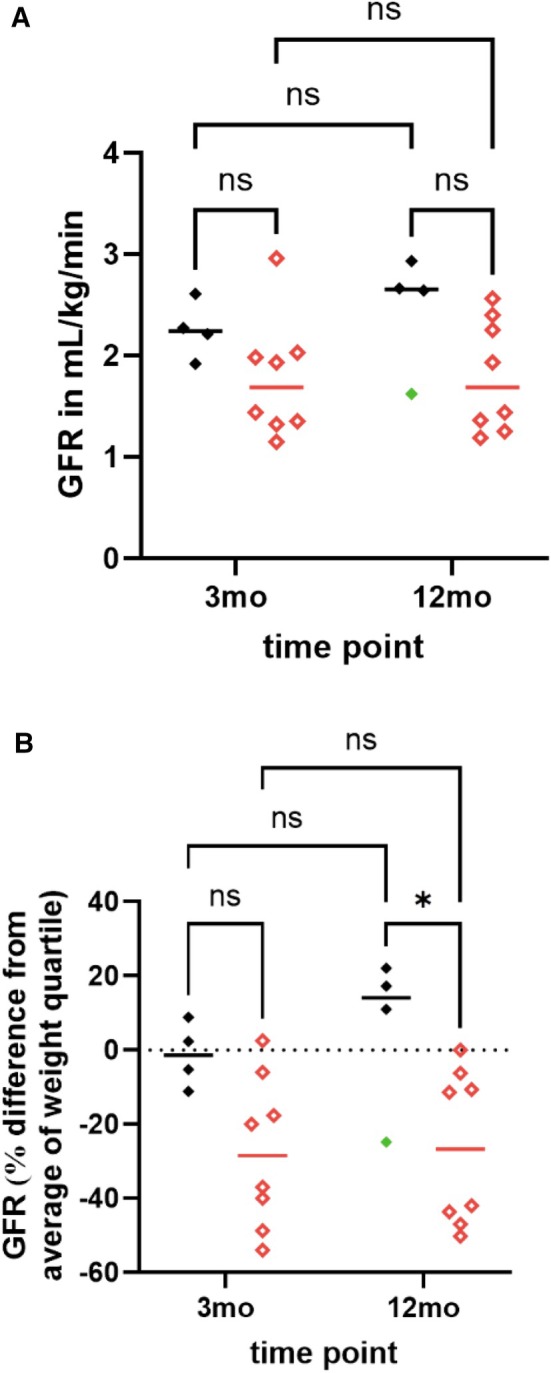
Absolute (A) and relative (B) changes in glomerular filtration rate over time in 12 dogs post AKI, per aetiology (leptospirosis in black vs. other causes in red) and per time point at 3 months (diamonds) and 12 months (squares). Data are presented as individual data points and median (horizontal line). For panel A, there was no significant difference between aetiology for absolute GFR at T2 (−0.34 mL/kg/min difference, 95% CI [−1.09 to 0.42], adjusted *P* = .42) or at T3 (−0.49 mL/kg/min, 95% CI [−1.24 to 0.27], adjusted *P* = .120). For panel B ** denotes significant difference in relative GFR between leptospirosis and other aetiologies at T3 (−45.2% difference, 95% CI [−18.7% to −71.5%], adjusted *P* < .025). No difference at T2 (−26.3% difference, 95% CI [−53.8% to 0.125%], adjusted *P* = .051). GFR Glomerular filtration rate, Mo Months.

Intraindividual difference in absolute GFR between T2 and T3 was +0.06 mL/kg/min (−0.59 to +0.72). Absolute GFR measurement increased in 8/12 (67%; 95% CI 35% to 90%) dogs between 3 and 12 months (Fig 4A, B, Table 4). The median USG for dogs where absolute GFR was unchanged or increased was 1.017 (1.009 to 1.053) at T2 (*n* = 9) and 1.038 (1.017 to 1.043) at T3 (*n* = 7). The USG of the dogs (*n* = 3) that had a decrease in absolute GFR between the time points was 1.15, 1.022 and 1.040 at T2 and 1.026, 1.029 and 1.030 at T3.

A significant difference in relative GFR was identified in dogs with AKI due to leptospirosis and AKI due to other aetiologies at T3 which was not identified at T2 (T3; −32.8% difference, 95% CI [−61.7% to −3.8%], adjusted *P*<.025, T2; −26.3% difference, 95% CI [−55.3% to 2.6%], adjusted *P*=.079, Fig 4B). Aetiology had no significant effect on SCr (T2; −33.6 μmol/L, 95% CI [−205 to 137], T3; −14.15 μmol/L, [95% CI −185 to 157], adjusted *P*=.672), SDMA (T2; −1.96 μg/dL, 95% CI [−6.18 to 2.26], adjusted *P*=.432, T3; −0.19 μg/dL, 95% CI [−4.41 to 4.03], adjusted *P* >.999) or absolute GFR (T2; −0.34 mL/kg/min difference, 95% CI [−1.09 to 0.42], adjusted *P*=.42, T3; −0.49 mL/kg/min, 95% CI [−1.24 to 0.27], adjusted *P* = .1996) at T2 and T3. Five dogs changed weight category between T2 and T3 (Table 4). Although the relative GFR changed, this change did not affect their classification of kidney dysfunction; three of these dogs had a decrease in relative GFR between T2 and T3. This reduction in GFR remained <20% of the mean of the body weight category in two dogs (Table 4: cases 5 and 6), and in one dog (Table 4: case 9) the GFR reduction was ≥20% below the mean of the body weight category and therefore classified as having kidney dysfunction despite having normal relative GFR at T2. This dog had received a 3‐month course of NSAIDs for an orthopaedic issue which had stopped 7 days prior to T3. In these three dogs, SCr and SDMA concentrations increased in one dog (Table 4: case 9) and two dogs, respectively (Table 4: cases 5 and 9).

## DISCUSSION

This study prospectively evaluated 15 dogs after AKI at standardised time points to determine the frequency of persistent azotaemia and progression of CKD post AKI. It also explored the role of GFR measurement, proteinuria and systemic hypertension to inform on CKD. Most dogs were non‐azotaemic over the follow‐up period, and dogs that survived had stable or improving renal function between 3 and 12 months. Measurement of GFR may have clinical utility in detecting and monitoring for persistently decreased kidney function in dogs recovering from AKI which may not be detected with routine markers of kidney function. However, monitoring trends in SCr and USG may be beneficial after AKI. Systemic hypertension and proteinuria were transient in most dogs with AKI.

Most dogs (10/15; 67%) were azotaemic at discharge, of which the majority were classified as IRIS grade I (5/15; 33%) and II (5/15; 33%) AKI, although 3/15 (20%) and 2/15 (13%) dogs, respectively, had grades III and IV AKI at discharge. The distribution of AKI grade at discharge was similar to a previous study that reported 25%, 30%, 30% and 15% dogs as IRIS AKI grades I, II, III, IV at discharge (Cambournac et al., 2019). Long‐term follow‐up was not reported in the previous study and therefore it is unknown whether resolution of azotaemia occurred as identified in the present study.

Although the majority of the dogs in our study were azotaemic at the point of discharge, the percentage of dogs with a SCr >144.5 μmol/L (1.6 mg/dL) at 3 months and 12 months was lower (20% and 14%, respectively). This occurrence of persistent azotaemia is lower than reported in other studies which have documented between 56% and 61% of dogs remaining azotaemic after AKI (Hetrick et al., 2022; Vaden et al., 1997). Although the aetiologies of AKI were similar in these studies (ischaemia, nephrotoxins and leptospirosis) comparisons are limited given that monitoring of SCr was not standardised. Hetrick et al. (2022) evaluated kidney outcomes in dogs with leptospirosis and reported a much higher occurrence of azotaemia after AKI (61%) but only 2/8 (25%) of these dogs had a SCr measurement >90 days after discharge. In people, the recovery phase of AKI is reported to be weeks to months and the term acute kidney disease is used to describe the loss of kidney function for a duration of between 7 and 90 days after exposure to AKI insult (Chawla et al., 2017; Kellum et al., 2012). It is therefore possible in the Hetrick et al. study (Hetrick et al., 2022) that the azotaemia in the other six dogs actually reflected acute kidney disease rather than CKD.

Based on serial SCr and SDMA measurements 10/15 (67%) and 4/15 (27%) of dogs were classified as IRIS CKD stage 1, 2 at 12 months and one dog that died before 12 months was classified as IRIS CKD stage 3. These results are consistent with previous clinical studies evaluating CKD stage outcomes ≥3 months after an AKI episode, although in these studies, the individual SCr concentrations and measurement time points were not specified (Bar‐Nathan et al., 2022; Ioannou et al., 2024). In the largest study (132 dogs), there was no difference in survival time between dogs that were azotaemic and those that were non‐azotaemic in a period of follow‐up between 30 days and 4.45 years (Bar‐Nathan et al., 2022). In our study, only one dog (that had been azotaemic at the time of discharge) died secondary to progression of kidney disease within 12 months. Further long‐term follow‐up of stage 2 and stage 3 CKD dogs post AKI is needed to better determine the association between persistent azotaemia and long‐term outcome.

Persistent kidney dysfunction, defined as a ≥20% decrease in GFR relative to body weight category, was detected in 4/12 (33%) and 5/12 (42%) dogs with a SCr <144.5 μmol/L (< 1.6 mg/dL) at T2 and T3. The relative reduction in GFR ranged from 24.8% to 54%. SDMA concentrations were similar in those dogs diagnosed with persistent kidney dysfunction and those with normal relative GFR at both time points. Therefore, despite SDMA being cited as a more sensitive marker than SCr for detecting a ≥40% decrease in GFR (Nabity & Hokamp, 2023), in this population, SDMA had limited utility in early detection of kidney dysfunction. Two out of 10 (20%) dogs classified as IRIS CKD stage 1, based on history and SCr and SDMA measurement had persistent kidney dysfunction based on GFR measurement. These results suggest that GFR measurement may have clinical utility at detecting persistent reduction in GFR in dogs recovering from AKI that cannot be identified with currently available surrogate markers of GFR. McKenna, Pelligand, Elliott, Walker, and Jepson (2020) demonstrated the clinical utility of GFR measurement in dogs with CKD and this is mirrored in our study. Such information may help decision‐making in terms of requirement for monitoring, avoidance of the use of nephrotoxic drugs or alteration in approach to therapies that may risk renal insult.

Within the IRIS guidelines SCr >125 μmol/L (>1.4 mg/dL) is used to differentiate the IRIS stage 1 and stage 2 boundary (Cowgill, 2023). In the present study, a persistent decline in kidney function was defined as a ≥20% decrease in GFR relative to body weight category. No dog with normal relative GFR had SCr >1.4 mg/dL (125 μmol/L) at either time point. Three out of four dogs and 2/5 dogs classified as having kidney dysfunction on GFR measurement at 3 and 12 months, respectively, had SCr >1.4 mg/dL (125 μmol/L). This result suggests monitoring trends in SCr may be beneficial after AKI and persistent elevation may alert the clinician to the potential for decreased kidney function. However, care, must be taken when interpreting single SCr concentrations and these measurements should be interpreted in light of breed, muscle condition score and hydration status (Cobrin et al., 2013).

Most dogs (10/12; 83%) that had GFR measurement performed had stable or improved kidney function between 3 and 12 months; this may be a result of intraindividual variation but may also suggest that the recovery phase of AKI continues beyond the 3‐month period.

In dogs that had GFR measurement and USG measurements available for review, the median USG was consistently higher at both time points in the dogs with normal relative GFR compared to those dogs with evidence of kidney dysfunction, although there was a degree of overlap. These results suggest that a persistent reduction in urine concentrating ability post AKI may raise the index of concern for renal dysfunction and may be an indication for GFR measurement.

Of the three dogs that had a decline in GFR between their 3‐ and 12‐month time points, the relative GFR remained within the normal range in two dogs. The final dog was exposed to a risk factor (NSAIDs) for AKI immediately prior to the 12‐month GFR assessment and showed a reduction in GFR (−0.59 mL/kg/min) in association with an increase in SCr between the two time points. Cowgill (2016) proposed that progressive CKD may result from overt or occult acute episodic or sustained insults to the kidney, such as the NSAID exposure this dog received. Continued follow‐up of these dogs exposed to risk factors such as medications/anaesthesia/dehydration using serial GFR estimation would be useful to explore this hypothesis further.

Despite many dogs having SBP ≥160 mmHg during hospitalisation, few required anti‐hypertensive therapy, and few dogs were hypertensive at follow‐up, suggesting the potential for transient causes of SBP ≥160 mmHg such as fluid overload. It does not appear in this population, unlike experimental canine studies or human studies, that SBP ≥160 mmHg was persistent nor associated with long‐term outcome (Finco, 2004). Although proteinuria was common in all dogs during hospitalisation, only one dog had proteinuria at one of the follow‐up examinations, and this was marginal (UPCR 0.52) suggesting that, in most dogs, proteinuria associated with AKI is transient. This differs from experimental canine and human studies and may reflect different underlying aetiologies for renal injury (Finco, 2004; Hsu et al., 2020).

An interesting finding of the study was the difference in the degree of kidney dysfunction between the different aetiologies of AKI. All the dogs diagnosed with leptospirosis (*n* = 4), had normal relative GFR at 3 months, and three dogs (excluding the dog that received a course of NSAID) demonstrated an increase in their GFR between 3 and 12 months with relative GFR being significantly higher than other aetiologies by 12 months. Conversely, those cases with a history of GA and/or NSAID administration had evidence of persistent kidney dysfunction even in those dogs who had normalised their SCr. These findings are similar to previous reports of long‐term outcomes in dogs with AKI; Legatti et al. (2018) reported a decreased 6‐month survival in AKI of non‐infectious aetiology compared to infectious aetiology and Bar‐Nathan et al. (2022) reported no dogs with AKI of an infectious aetiology, predominantly leptospirosis, died during follow‐up.

The main limitation of this study was the small population size, with limited aetiologies of AKI and restriction to 12‐month follow‐up. It therefore remains unknown if those dogs with IRIS CKD stage 1 kidney disease would demonstrate progressive CKD in the future or whether there are risk factors from the time of AKI that may predict this eventuality. Although GFR measurement was performed on serial occasions, baseline GFR measurement was not performed. However, 10 of the dogs were still azotaemic at the time of discharge indicating kidney dysfunction was present at that time. Absolute and relative GFR measurements were reported and compared between aetiologies and both time points as well as compared between time points in an individual. Interpretation of isolated GFR measurements can be difficult, particularly in light of intraindividual variation in GFR. Individual variability for GFR estimation via iohexol clearance has previously been reported as 19.9% for non‐azotaemic cats (Finch et al., 2018). For this study, a cut‐off of ≥20% below the mean of the body weight category was used to support kidney dysfunction; however, it is unclear whether intraindividual variation in GFR in dogs is the same as in cats. Relative GFR was calculated based on body weight category and five dogs changed their body weight category between visits. In one dog, this change in body weight category between visits resulted in a change in relative GFR despite absolute GFR not differing between visits. This highlighted the importance of assessing absolute change in GFR as well as reviewing change in body weight category between visits for individual dogs when interpreting relative GFR.

In conclusion, in this population of dogs surviving more than 3 months post AKI, persistent azotaemia occurred infrequently. However, GFR measurement was able to identify a clinically relevant reduction in GFR in those dogs that had returned to a non‐azotaemic state and therefore its likely care should be taken with the use of nephrotoxic drugs in dogs with a history of AKI, even if their SCr has normalised. Persistent proteinuria and SH after AKI in this population of dogs were uncommon and therefore their effect on outcome remains unknown. Further work is required to determine the longer term outcome of those non‐azotaemic dogs that continued to show decreased kidney function on the basis of their GFR assessment and to understand whether alteration in management might impact outcome in these dogs. Larger studies are required to explore the impact of aetiology on long‐term kidney function.

### Author contributions

**L. P. Cole** collected the samples, performed the study, analysed the data, interpreted the results, and wrote the first draft of the manuscript. **L. Pelligand** contributed to study design, analysed results and critically reviewed the manuscript. **R. Jepson** and **K. Humm** contributed to study design and critically reviewed the manuscript. All authors read and approved the final manuscript.

### Conflict of interest

Ludovic Pelligand has affiliation with deltaDOT through a Concept Development Partnership, which resulted in the employment of a postdoctoral researcher for 4 years for the development of the GFR service.

## Data Availability

The data that support the findings of this study are available from the corresponding author upon reasonable request.
